# A Few-Shot SE-Relation Net-Based Electronic Nose for Discriminating COPD

**DOI:** 10.3390/s25154780

**Published:** 2025-08-03

**Authors:** Zhuoheng Xie, Yao Tian, Pengfei Jia

**Affiliations:** 1 School of Mechanical Electrical and Information Engineering, Shandong University, Weihai 264209, China; 202200800061@mail.sdu.edu.cn; 2 School of Future Technology, Xi’an Jiaotong University, Xi’an 710049, China; tianyaochina@stu.xjtu.edu.cn; 3 State Key Laboratory for Manufacturing Systems Engineering, Xi’an Jiaotong University, Xi’an 710049, China; 4 School of Electrical Engineering, Guangxi University, Nanning 530004, China; 5 Guangxi Key Laboratory of Intelligent Control and Maintenance of Power Equipment, Guangxi University, Nanning 530004, China

**Keywords:** chronic obstructive pulmonary disease (COPD), electronic nose, few-shot gas classification, relation network, lung cancer

## Abstract

We propose an advanced electronic nose based on SE-RelationNet for COPD diagnosis with limited breath samples. The model integrates residual blocks, BiGRU layers, and squeeze–excitation attention mechanisms to enhance feature-extraction efficiency. Experimental results demonstrate exceptional performance with minimal samples: in 4-way 1-shot tasks, the model achieves 85.8% mean accuracy (F1-score = 0.852), scaling to 93.3% accuracy (F1-score = 0.931) with four samples per class. Ablation studies confirm that the 5-layer residual structure and single-hidden-layer BiGRU optimize stability (h_F1-score ≤ 0.011). Compared to SiameseNet and ProtoNet, SE-RelationNet shows superior accuracy (>15% improvement in 1-shot tasks). This technology enables COPD detection with as few as one breath sample, facilitating early intervention to mitigate lung cancer risks in COPD patients.

## 1. Introduction

Lung cancer is a highly lethal malignancy that originates from bronchial mucosa or glands. Only 15% of patients are diagnosed in the early stage, while 85% are diagnosed at an advanced stage [1,2,3]. Late diagnosis is often due to similar symptoms with viral diseases, leading to a high mortality rate of around 90% [4]. Early detection is crucial in reducing mortality rates, making cancer prevention and detection significant research topics.

Chronic obstructive pulmonary disease (COPD) is one of the top ten non-infectious diseases worldwide. It is a chronic inflammatory lung disease that causes obstruction in the airflow from the lungs [5]. Research indicates that approximately 1% of COPD patients develop lung cancer annually [6]. Moreover, published studies [7,8] have demonstrated that COPD can serve as a critical and reliable predictor for screening lung cancer risk. The validity of this finding was confirmed by the COPD Lung Cancer Screening Score (COPD-LUCSS), which exhibited a strong correlation between COPD and lung cancer risk.

Several methods have been proposed for testing chronic obstructive pulmonary dis-ease (COPD), including gas chromatography–mass spectrometry (GC-MS) [9,10], spirometry [11,12,13], sputum cytometry, chest radiography [14], and fluoroscopic bronchoscopy. Despite their established roles, each method presents significant limitations. These drawbacks include requirements for specialized personnel, complex and time-consuming procedures, high associated costs, and in some cases, patient invasiveness. Recently, electronic nose (E-nose) applications have emerged as a promising alternative, offering a comparatively easy and fast approach for detecting COPD [15]. Table 1 summarizes the key characteristics and limitations of the mentioned COPD detection methods compared to the emerging electronic nose technology.

The electronic nose (E-nose) is a tool designed to simulate the structure and operation of the human nose, which can assist or replace humans in gas research [16]. It comprises three parts: a sensor array, signal processing, and pattern recognition, which together simulate the biological olfactory system’s response to odor. In recent years, e-noses have supported remarkable achievements in medical diagnosis [17,18,19], environmental monitoring [20,21], food safety [22,23,24], and intelligent agriculture [25,26,27]. Selecting appropriate sensors can ensure that the electronic nose accurately captures the signal characteristics of the target gas [28], while designing excellent pattern recognition algorithms can efficiently utilize these features to significantly enhance the performance of the electronic nose [29]. To enable e-noses to have anthropomorphic or even surpass human gas analysis capabilities, researchers must continuously explore effective gas-recognition algorithms to adapt to various research contexts. In the field of gas classification and concentration prediction, the feasibility of traditional machine learning models such as support vector machine (SVM) [30], XGBoost [31], kernel principal component analysis (KPCA) [32], random forest [33], and deep learning models such as convolutional neural network (CNN) [34] and long short-term memory (LSTM) [35] has been demonstrated one after another.

Current research on e-noses tends to combine long-term data collection with machine learning or deep learning for high performance, although this requires a large number of gas samples for training and a fixed e-nose device. Using too few samples for training can cause overfitting, while using a different sensor array risks bias in the results obtained from the model trained on the old device. Collecting a large number of breath samples is costly for medical institutions, and models trained with many samples can only be used on a specific device, making widespread e-nose use for COPD detection challenging.

Humans can learn new things quickly and accurately with only a few samples. For instance, a person who smells jasmine once can recognize the scent very easily due to their prior knowledge from other experiences. The idea behind FSL is to leverage prior knowledge and train a model with only a small number of samples when faced with a new task. FSL can solve problems such as traditional machine learning algorithms overfitting due to limited data and the inability to directly use deep neural networks and other algorithms that require large amounts of labeled data due to difficulties in labeling or noise. In recent years, FSL based on metric learning has rapidly developed in computer vision and natural language processing. The strategy is to learn prior knowledge to calculate similarities between any two samples and classify unknown samples. Various methods have been proposed, including siamese networks (SiameseNets) [36], matching networks (MatchingNets) [37], prototypical networks (ProtoNets) [38], and Relation Networks (RelationNets) [39], achieving better results in different tasks. Using FSL for e-nose detection enables the device to determine if a new sample is from a COPD patient by training a deep learning network with only a small number of breath samples, promoting the application of e-nose detection for COPD.

In this paper, we construct a model called SE-RelationNet based on the idea of metric learning, and the research performed is as follows: (1) Construct a relational network with residual blocks and bi-directional gate recurrent unit (BiGRU) blocks as the main body, and add squeeze–excitation blocks (SEblock) to improve the performance of the model. (2) Learning prior knowledge from a common gas dataset and solving the problem of detecting patients’ breath in COPD under small-sample situations.

The article outlines the experimental configuration and gas-sampling method in Section 2, introduces the proposed SE-RelationNet in Section 3, discusses the performance of the experiments in Section 4, performs ablation and contrast experiments in Section 5, and concludes in Section 6.

## 2. Materials and Environments

FSL requires learning problem-solving experience from prior knowledge, with the dataset providing this knowledge referred to as the meta-training set and the dataset used for solving the problem known as the meta-testing set. To ensure compatibility with the same neural network, samples from both sets need to be transformed into the same format before being fed into the model. Generally, the meta-training set should contain more categories to represent prior knowledge, and the meta-training and meta-testing sets should strongly correlate for better transferability [40].

To tackle the challenge of screening COPD patients using only a limited number of labeled breath samples, appropriate selection of the meta-training and validation sets is crucial. Selection criteria include the use of a gas sensor-array collection, with each sample containing the process of starting and stopping the flow of the target gas, and the transformation of both datasets into the same shaped matrix during preprocessing. We chose the “Gas sensor arrays in open sampling settings Data Set” [41] as the meta-training set, containing samples of over 10 common gases collected under normative conditions. Meanwhile, we selected the “Electronic nose dataset for COPD detection from smokers and healthy people through exhaled breath analysis,” including a range of breath samples from COPD patients, general population, smokers, and air samples [42]. These samples can simulate scenarios with very few labeled samples (1–4 per class), where traditional machine learning methods may lead to overfitting. Thus, small-sample learning methods are required in such cases. The two datasets have different collection methods, data formats, and sensor arrays, demonstrating the generality of the model with less stringent requirements for sensor arrays and collection methods. In the following, we will briefly describe these two datasets and illustrate the preprocessing methods.

### 2.1. Meta-Training Set

The meta-training dataset was sourced from the UCI Machine Learning Repository and was curated by Alexander Vergara et al. For a comprehensive understanding, please refer to paper [43]. This dataset was gathered using an array of 72 sensors, organized into nine groups, within a turbulent wind-tunnel environment infused with various gases. To identify the most discriminative sensors, we implemented a mutual information (MI)-based feature-selection approach evaluating both static and dynamic response characteristics. Mutual information quantifies the dependency between sensor features and gas categories. For a discrete feature *X* (e.g., *X*_mean_ or *X*_slope_ and class label *Y*, it is computed as:

(1) $$\mathrm{MI}(X;Y)=\sum_{y\in Y}\sum_{x\in X}p(x,y)\log\frac{p(x,y)}{p(x)p(y)}$$

where *p*(*x*,*y*) is the joint probability distribution, and *p*(*x*), *p*(*y*) are marginal distributions. Higher MI values indicate stronger relevance for classification. Continuous features were discretized using histogram binning (20 bins) to enable probability estimation. For each sensor, temporal average response *X*_mean_) captured static properties while maximum instantaneous slope (*X*_slope_ = max |∇ data|) quantified dynamic sensitivity. Sensors were ranked by combined MI score ${\mathrm{MI}}_{\mathrm{combined}}$, with the top eight sensors selected based on their discriminative power for gas classification tasks. The ${\mathrm{MI}}_{\mathrm{combined}}$ is computed as:

(2) $${\mathrm{MI}}_{\mathrm{combined}}=\frac{1}{2}\left(\mathrm{MI}(X_{\mathrm{mean}},Y)+\mathrm{MI}(X_{\mathrm{slope}},Y)\right)$$

Detailed information regarding the placement, type, contribution score, and specific gas sensitivities of these sensors is provided in Table 2.

The arrangement of the study involved altering the horizontal distance between the gas source and the sensor array. Data was collected for 11 different gases at six varying distances, with the sensors functioning under five distinct operational conditions. Additionally, the wind speed within the tunnel was modified to three separate levels. Each experimental configuration was subjected to 20 repetitions, and for each repetition, sensor data was logged at a 10 ms interval across a total duration of 400 s, resulting in 40,000 data points per sample.

**Figure 1:**
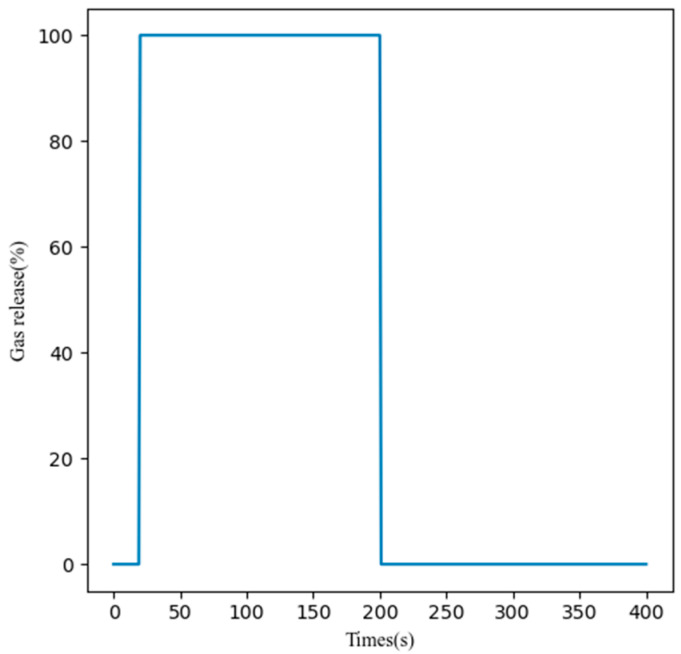
Gas release rate in wind tunnel over time during meta-training set collection.

To ensure stable sensor readings, the wind tunnel was initially flooded with pure air for the first 20 s. Subsequently, the experimental gas was introduced from the 20 s mark until 200 s. From 200 to 400 s, the tunnel was once again ventilated with pure air to cleanse the system. A visual representation of the gas release over time is depicted in Figure 1. Through this methodology, a comprehensive dataset comprising 1800 samples for each gas type was amassed.

During the preparation of the meta-training set, samples with missing data were discarded to maintain the integrity of the dataset. This step was crucial to align the shape of the meta-training set with that of the meta-testing set for effective few-shot learning (FSL). To condense the data, the recordings from each sensor were averaged per second, resulting in 100 data points per second. This reduction led to each sample being represented as a [400, 8] matrix, reflecting 400 s of data from eight sensors. To extract the most pertinent time-series information, the data window was further narrowed down to the segment spanning from 17 to 257 s. This truncation provided a refined sample representation in the form of a [240, 8] matrix, capturing the essential trends in sensor response.

The finalized meta-training set includes 11 gas classes, each uniquely identified by name, molecular formula, concentration, and the number of samples collected, as listed in Table 3.

### 2.2. Meta-Testing Set

Our meta-testing set was obtained from Mendeley Data, collected and produced by Cristhian Manuel et al. Refer to their paper for more details. The dataset was created to aid the diagnosis of chronic obstructive pulmonary disease and contains four categories of samples: breath of COPD patients (COPD), breath of smokers (SMOKERS), breath of healthy people who do not smoke (CONTROL), and air (AIR). The number of samples in each category is shown in Table 4.

**Figure 2:**
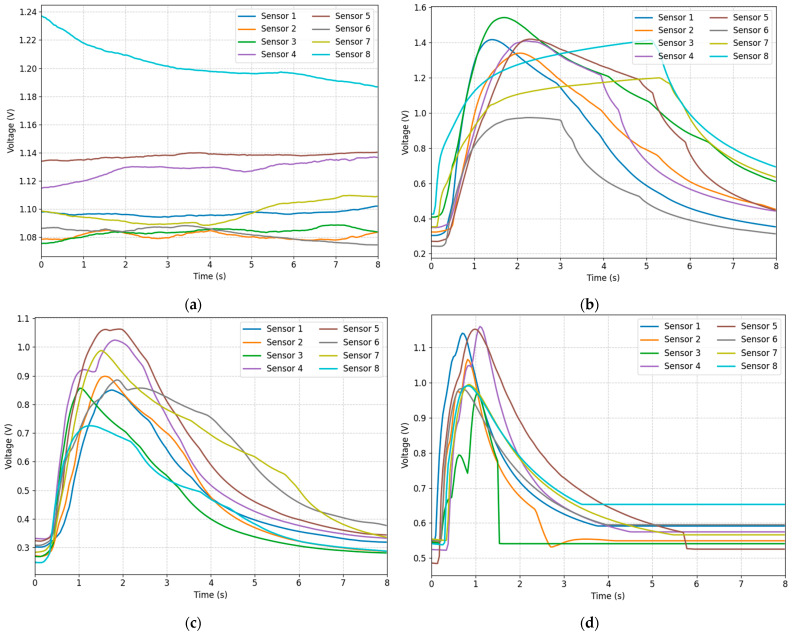
Average voltage values recorded by each sensor for each class of breath: (**a**) Air; (**b**) Control; (**c**) COPD; and (**d**) Smokers.

A sensor array of eight sensors was used, with each sample collection involving the subject blowing into the gas while the sensors collected 500 sets of data per second for a total duration of 8 s. This resulted in a matrix of shape [4000, 8], which provided a more complete picture of the approximate trend of the sensor response changes. For each type of sample, we drew a variation curve of the average value of the response of each sensor, as shown in Figure 2.

To ensure that the meta-testing set had the same sample shape as the meta-training set, we used the equal time-interval extraction method to extract 240 out of 4000 recording points. This allowed us to obtain a matrix with a shape of [240, 8].

### 2.3. Experimental Environment

This experiment was conducted using Python v3.9 and implemented on the PyTorch (v1.13.0) deep learning framework. All models were computed using CUDA with a version of 11.7 for optimized performance.

### 2.4. Signal Preprocessing

To enhance the robustness and generalization ability of the model, we performed the following signal-preprocessing steps:

(1) Normalization: First, we normalized all sensor data to make them have the same scale. This eliminates the differences in response intensities between different sensors and makes the model more sensitive to the range of input data.
(2) Channel Shuffling: During each training round, we randomly shuffle and rearrange the sensor channels for all samples within the batch. This aims to prevent the model from over-relying on a specific channel order, thereby enhancing its ability to calculate similarity under different channel orders. Essentially, this is a data augmentation technique that increases the number of training samples and enables the model to learn more generalizable feature representations.

## 3. Methodology

In this section, we introduce the SE-RelationNet which comprises an embedding module $f_{\phi }$ and a metrics module $g_{\varphi }$. The embedding module extracts sample features using a deep network structure, while the metrics module calculates similarity between two feature matrices. Section 3.1 explains how to use this network for few-shot learning, Section 3.2 covers the embedding module, and Section 3.3 discusses the metrics module.

### 3.1. Training Method of SE-RelationNet

**Figure 3:**
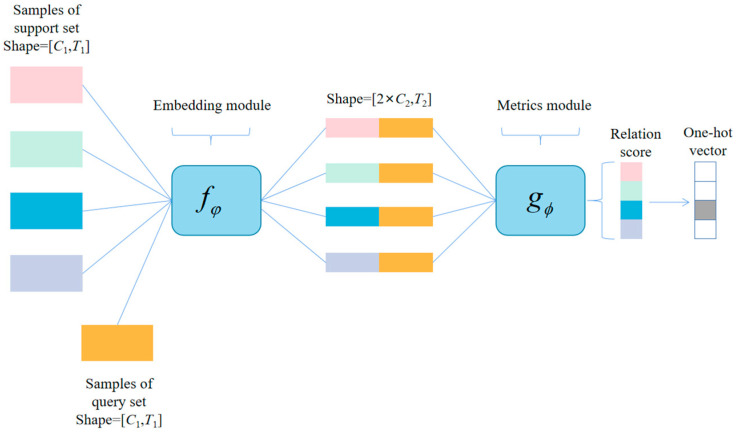
The overall structure of the SE-RelationNet.

The overall structure of the SE-RelationNet is illustrated in Figure 3. To tackle the few-shot classification task, the model is trained using the *N*-way *K*-shot method. Specifically, *N* classes are selected and *K* samples are randomly drawn from each class to construct the support set $S={\left\{\left(x_{i},y_{i}\right)\right\}}_{i=1}^{m}(m=K\times N)$. Then, *P* samples are drawn from the remaining part of each category to construct the query set $Q={\left\{\left(x_{j},y_{j}\right)\right\}}_{j=1}^{n}(n=P\times N)$. The training process for *K* = 1 and *K* > 1 is explained separately.

i. *K* = 1. Few-shot learning with *K* = 1, also known as one-shot learning, involves processing a sample $x_{j}(j=1,2,\ldots ,P\times N)$ in the query set using a sample $x_{i}(i=1,2,\ldots ,N)$ in the support set through an embedding module $f_{\phi }$ to obtain features. The feature-merging operator $C\left(-,-\right)$ then combines the obtained features, resulting in input $C\left(x_{i},x_{j}\right)$ for the metrics module $g_{\varphi }$. This generates a similarity score $c_{i,j}$ between 0 and 1, which represents the similarity of $x_{i}$ with $x_{j}$.


(3) $$c_{i,j}=g_{\phi }(C(f_{\phi }(x_{i}),f_{\phi }(x_{j}))),i=1,2,\ldots ,N$$

ii. *K* > 1. In the *K*-shot case with *K* > 1, the embedding module averages the samples of each class in the support set to obtain the features for that class. The resulting features for each class are then combined with the samples in the query set and input into the metrics module. The metrics module then outputs similarity scores between the samples in the query set and each class of samples in the support set.

To train the model, we use mean square error (MSE) loss, which is typically used for regression problems that resemble classification problems in the label space {0,1}. However, since our model predicts similarity scores, the problem can also be viewed as a regression problem, as shown in the following equation:

(4) $$\phi ,\varphi \leftarrow \underset{\phi ,\varphi }{\arg\min}\sum_{i=1}^{m}\sum_{j=1}^{n}{\left(r_{i,j}-1(y_{i}==y_{j})\right)}^{2}$$

Once the model is trained with multiple randomly generated tasks, it can determine the degree of similarity between any two samples to a certain extent. During the testing session, the N-way K-shot task is performed several times on the meta-testing set. The class with the highest degree of similarity to the unknown class of samples is selected as the class for that sample, and the model is evaluated using metrics such as accuracy rate.

### 3.2. Embedding Module

**Figure 4:**
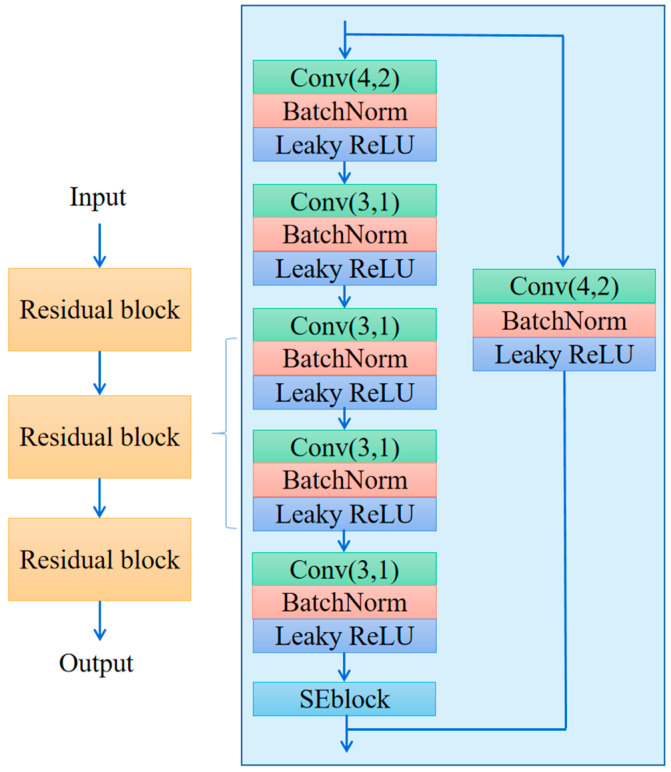
The structure of the embedding module, with residual blocks in a line in the blue box.

The embedding module is the first module through which the sample data passes. Whether it is a meta-training set sample or a meta-testing set sample, it is in the form of a matrix with the shape [240, 8], representing the data recorded by 8 sensors at 240 recording points. The embedding module extracts abstract features from this time series for further processing by the metrics module. Its output is a matrix with the shape [63, 30]. Figure 4 depicts the structure of the embedding module.

The ability of a neural network to extract abstract features improves with increasing depth. However, a network that is too deep can suffer from gradient dispersion and gradient explosion. Traditional solutions such as normalized initialization and batch normalization may slow down the original problem to some extent, but they introduce new problems. One of these problems is the degradation of network performance. Kaiming He proposed residual blocks as an effective solution to this problem. Hence, we added three residual blocks to our network [44].

Each residual block comprises pathway $F_{1}$ and pathway $F_{2}$. Pathway $F_{1}$ includes three convolutional layers and one SEblock, while pathway $F_{2}$ consists of only one convolutional layer. Assuming that *x* is the input of the residual block and *y* is the output, $w_{1}$ and $w_{2}$ are the parameters of pathway $F_{1}$ and pathway $F_{2}$, respectively, which are also the objects we need to optimize. The equation below shows the relationship between the input and output:

(5) $$y=F_{1}(x,\left\{w_{1}\right\})+F_{2}(x,\left\{w_{2}\right\})$$

**Figure 5:**
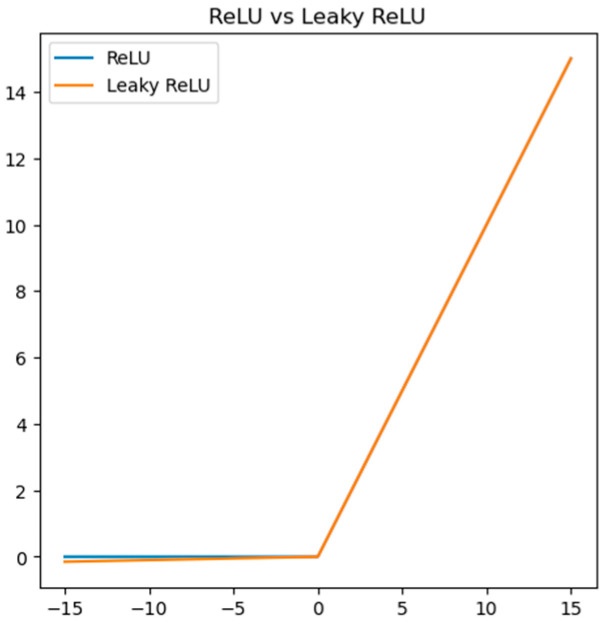
Comparing the shapes of leaky ReLU and ReLU functions.

During backpropagation, gradient fading may occur if the pathway between layers is too long. However, using a shorter pathway $F_{2}$ can mitigate this issue by propagating gradients across fewer layers. Leaky ReLU is a modified linear activation function with *f*(*x*) = max (*ax*, *x*), where *a* < 1 (usually set to 0.01). It has better convergence and generalization capabilities compared to traditional ReLU and can improve the accuracy and stability of deep neural networks. When a value of 0.01 is used for parameter *a*, the shapes of the leaky ReLU function and the ReLU function can be plotted as illustrated in Figure 5.

**Figure 6:**
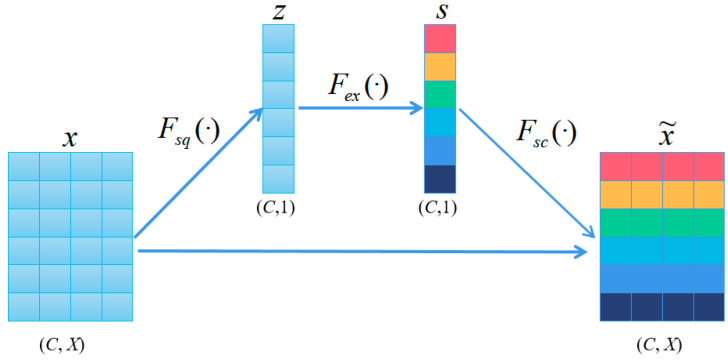
Structure of SEblocks.

In the network, we incorporated the SEblock, an attention mechanism illustrated in Figure 6, to enhance its performance [45]. The SEblock selectively emphasizes informative features by adaptively recalibrating them based on their relevance. The basic idea is as follows:

(1) Squeeze (*F_sq_*). Aggregates the features of each channel by averaging pooling:

(6) $$z_{c}=F_{sq}(x_{c})=\frac{1}{X}\sum_{i}^{T}x_{c}(i)$$

Here, *z_c_* is the compressed channel vector, *x_c_* is the *c*-th channel of the input feature map, and *T* is the dimension of each channel.

(2) Extraction (*F_ex_*). The compressed vectors undergo two fully connected layers to produce channel weights. To improve computational efficiency, we set a reduction factor ratio and halve the number of neurons in the first layer by $\frac{1}{ratio}$ while using ReLU as a nonlinear function. The second layer has the same number of neurons as the input and applies the sigmoid function to confine the weights between 0 and 1. These fully connected layers are parameterized by $w_{1}^{\prime }$ and $w_{2}^{\prime }$.

(7) $$s=F_{ex}(z)=f_{2}(f_{1}(z,\left\{w_{1}^{\prime }\right\}),\left\{w_{2}^{\prime }\right\})$$

Here, *f*_1_ and *f*_2_ are two consecutive fully connected layers used to process. This step enables SEblocks to use the global information of each channel and selectively emphasize the channel features.

(3) Scale (*F_sc_*). The importance score of each channel is obtained from the “Extraction“ stage, which we use to reweight the channels. This involves sequentially multiplying each channel with its corresponding weight to produce the calibrated attention channels.

(8) $${\tilde{x}}_{c}=F_{sc}(x_{c},s_{c})=s_{c}\cdot x_{c}$$

### 3.3. Metrics Module

**Figure 7:**
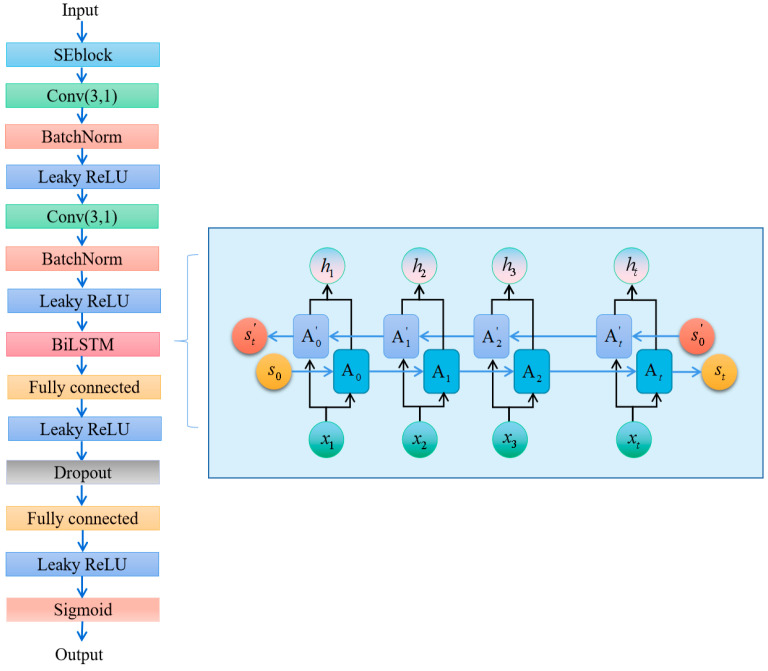
Structure of metrics module with BiGRU block in blue box.

To obtain the similarity between unknown and known category samples, we concatenate them and input them into the “metrics” module illustrated in Figure 7. The metrics module consists of a convolutional layer for abstract feature extraction, BiGRU blocks (a variant of GRU for time-series feature extraction), SEblock for improved expressiveness, and a fully connected layer to process the output data from the BiGRU block. Finally, the sigmoid function is applied to the output to produce a probability score between 0 and 1, indicating the predicted category of the unknown sample.

The BiGRU block, illustrated in Figure 7, introduces the concept of hidden state to extract time-series features by learning the information at each moment and combining it with the information before and after [46]. Compared to traditional fully connected layer networks, this results in improved feature-extraction performance. The input–output relationships for each layer can be expressed as follows:

(9) $$r_{t}=f(W_{r}x_{t}+U_{r}h_{t-1}+b_{r})$$

This calculates the “reset gate” value. It decides how much of the past hidden state *h_t_*_−1_ to forget or reset based on the current input *x_t_*. A value close to 0 means discarding most past information, while a value close to 1 means retaining it. This helps the model ignore irrelevant historical data when processing new inputs.

(10) $$z_{t}=f(W_{z}x_{t}+U_{z}h_{t-1}+b_{z})$$

This computes the “update gate” value. It determines how much new information from the current input should update the hidden state. For example, if *z_t_* is near 1, the hidden state relies heavily on past values; if near 0, it prioritizes new inputs. This gate balances between retaining long-term memory and incorporating fresh data.

(11) $$h_{t}^{\prime }=\tan h(W_{h}x_{t}+U_{h}(r_{t}⊙h_{t-1})+b_{h})$$

This generates a “candidate” for the new hidden state. It combines the current input *x_t_* with a filtered version of the past hidden state (using the reset gate *r_t_*). The tanh function ensures the output is normalized, preventing extreme values. Essentially, this step proposes a new state based on selective past and current information.

(12) $$h_{t}=z_{t}⊙h_{t-1}+(1-z_{t})⊙h_{t}^{\prime }$$

This produces the final hidden state *h_t_* by blending the previous hidden state *h_t_*_−1_ and the candidate $h_{t}^{\prime }$, using the update gate *z_t_* as a weighting factor. If *z_t_* is high, the state leans toward history; if low, it favors the new candidate. This allows the BiGRU to adaptively learn sequential patterns, such as trends in breath sample responses.

To optimize model performance, we set the number of hidden layers in the BiGRU block to 1.

## 4. Experiments and Analysis

In this section, we begin by setting appropriate parameters for the SE-RelationNet and assessing its performance.

### 4.1. Parameter Optimization of the SE-RelationNet

SE-RelationNet’s training benefits from setting optimal parameters for improved accuracy and faster convergence. The trial-and-error method is used to select the best parameters, which are listed in Table 5 for easy replication of the model.

### 4.2. Selection of Evaluation Indicators

To evaluate the SE-RelationNet model, we used four metrics: *mean_accuracy*, *h_accuracy*, *mean_F*1*-score*, and *h_F*1*-score*. Additionally, we employed a confusion matrix as a common visualization tool for supervised learning, which can be seen in Table 6.

The formula for *accuracy*, which is the most commonly used evaluation metric for classification tasks, is as follows:

(13) $$accuracy=\frac{TP+TN}{TP+TN+FP+FN}$$

The *F*1*-score* is a reconciled mean of *precision* and *recall*, proposed as a more robust indicator than *accuracy* due to susceptibility to sample equilibrium. Its formula is:

(14) $$precsion=\frac{TP}{TP+FP}$$

(15) $$recall=\frac{TP}{TP+FN}$$

(16) $$F1-score=\frac{2\times prcesion\times recall}{precsion+recall}$$

For each round the model is trained, we will test the model using a meta-test set, and each test yields *accuracy* and *F*1*-score*. Let the *n* different *accuracy*’s obtained be $a_{1}$, $a_{2}$, …, $a_{n}$, and the *n* different *F*1*-scores* are $f_{1}$, $f_{2}$, …, $f_{n}$. To accurately measure the model effect, we take the average of these two metrics separately:

For each training round, the model is tested on a meta-test set to obtain *accuracy* and *F*1*-score*. Let the *n* different accuracy scores be $a_{1}$, $a_{2}$, …, $a_{n}$, and the *n* different *F*1*-scores* be $f_{1}$, $f_{2}$, …, $f_{n}$. To accurately measure the model’s effectiveness, we calculate the average of these two metrics separately:

(17) $$mean\_accuracy=\frac{1}{n}\sum_{i=1}^{n}a_{i}$$

(18) $$mean\_F1-score=\frac{1}{n}\sum_{i=1}^{n}f_{i}$$

To assess the dispersion of $a_{1}$, $a_{2}$, …, $a_{n}$, we assume that they follow a t-distribution and introduce the distance *h_accuracy*, which represents the distance between the right endpoint of the 95% confidence interval and the *mean_accuracy*. The formula for calculating *h_accuracy* is as follows:

(19) h_accuracy=sem⋅x

(20) $$sem=\frac{\sigma }{\sqrt{n}}$$

where *sem* and σ are the standard error and standard deviation of *n accuracy*, respectively, and *x* are the positions of the right end quantile of the *t* distribution at a confidence level of 95% and a degree of freedom of *n* − 1. *h_accuracy* is calculated in the same way as *h_F*1*-score*. When *mean_accuracy* and *mean_F*1*-score* are larger, the stronger the ability of the model to predict correctly. The smaller the *h_accuracy* and *h_F*1*-score* values, the higher the confidence level of the model and the less randomness present in the model performance due to training.

**Figure 8:**
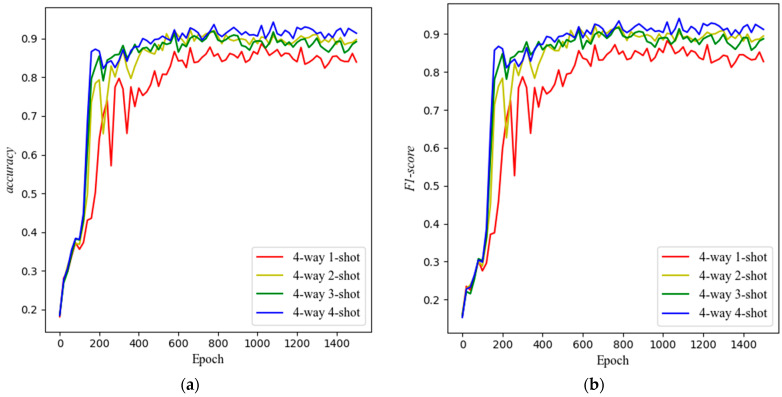
(**a**) *Accuracy* with epochs in meta-testing set testing. (**b**) *F*1*-score* variation with epochs in meta-testing set testing.

For every 20 epochs trained by the model, we conduct a test containing 50 epochs on the meta-testing set (each epoch consisting of randomly divided *N*-way *K*-shot tasks) to obtain *accuracy* and *F*1*-score*. Figure 8 shows a line graph with epoch as the horizontal axis and *accuracy* or *F*1*-score* as the vertical axis. The graph indicates that both metrics exhibit an overall upward trend until the 600th epoch, after which they fluctuate around a certain value.

Select the records between 700 and 1000 epochs with *accuracy* and *F*1*-score* to find their indicators as shown in Table 7.

Table 7 shows that *mean_accuracy* and *mean_F*1*-score* increase with increasing *K* when there is no clear trend in the metrics. However, this increasing trend is not significant when *K* is greater than 1. This suggests that increasing *K* within a certain range can improve the model’s effectiveness, but using a larger *K* implies a larger sampling scale, which may not necessarily lead to better results beyond a certain point.

The marginal improvement from *K* = 1 to *K* = 4 stems from the inherent properties of the learned embedding space and the metric mechanism. Our model focuses on extracting abstract feature representations (or “class prototypes”) through the embedding module, with *K* primarily influencing the robustness of prototype construction during metric comparison.

(1) *K* = 1 performance: The strong baseline accuracy (e.g., >0.85 *mean_F*1*-score* in 4-way tasks) indicates effective generalization, as a single sample suffices to capture core class characteristics. However, individual sample noise or outliers can degrade prototype fidelity.
(2) *K* > 1 refinement: Increasing *K* averages out noise and incorporates diverse sample variations, enhancing prototype stability. This explains the gradual accuracy rise up to *K* = 4.
(3) Asymptotic behavior beyond *K* = 4: Once *K* exceeds a threshold (~4 in our experiments), prototypes saturate in representational quality. Further samples yield diminishing returns, as the embedding space already encodes class-discriminative features efficiently.

This phenomenon mirrors human cognition: recognizing a new object after one exposure (*K* = 1) is possible but error-prone; repeated exposures (*K* > 1) refine mental prototypes until stability is achieved. Thus, the limited *K*-scaling gain validates the embedding space’s optimality—a few samples suffice for near-peak generalization.

## 5. Results and Discussion

In this section, we performed ablation experiments to identify the optimal number of residual block layers, assess the suitability of BiGRU blocks, and evaluate the effectiveness of the attention mechanism. Additionally, we included two few-shot learning models as controls for the proposed model.

### 5.1. Making Changes to the BiGRU Block

To investigate the rationality of the BiGRU with a single hidden layer, we designed control experiments for the BiGRU. The control group replaced the BiGRU with one hidden layer (group 1) with a BiGRU with two hidden layers (group 2), a BiGRU with three hidden layers (group 3), a GRU block with one hidden layer (group 4), a BiLSTM block with one hidden layer (group 5), a RNN block with one hidden layer (group 6), and a LSTM block with one hidden layer (group 7), respectively. To ensure the evaluation reflects the model’s performance at convergence, records between 700 and 1000 epochs were selected for aggregation. This interval was chosen because, as illustrated in Figure 8, the F1-score for all models, particularly SE-RelationNet, exhibited minimal fluctuations and stabilized after approximately 700 training epochs. The calculated mean_F1-score and h_F1-score within this stable period provide a reliable assessment of the model’s generalization capability, as shown in Table 8.

The results show that the BiGRU block with one hidden layer (group 1) consistently outperformed deeper variants (groups 2 and 3) across all few-shot settings (Table 7). This superiority arises primarily from reduced overfitting risk and computational efficiency. Deeper networks introduce more parameters, making them prone to memorizing noise rather than learning generalizable features in the limited-data context of our meta-testing set. The single-layer architecture avoids this degradation and manages gradients more effectively. Furthermore, the bidirectional design (group 1) significantly outperformed the unidirectional GRU (group 4), particularly in the challenging 1-shot task (0.852 vs. 0.816 mean_F1-score), due to its enhanced contextual awareness by processing sequences in both forward and backward directions, capturing complex temporal patterns in sensor responses (Figure 2). While BiLSTM (group 5) showed competitive performance in some tasks (e.g., 4-way 4-shot), the GRU-based model (group 1) generally achieved higher or comparable mean_F1-scores (e.g., 0.852 vs. 0.823 in 4-way 1-shot), making it better suited for our small-sample learning scenario. Therefore, BiGRU with one hidden layer is the best design.

The results show that the BiGRU block with one hidden layer and the convolutional layer outperformed the other structures, but the former showed higher stability compared to the latter. Overall, the BiGRU block with one hidden layer is the better design.

### 5.2. Selection of the Number of Residual Block Layers

**Figure 9:**
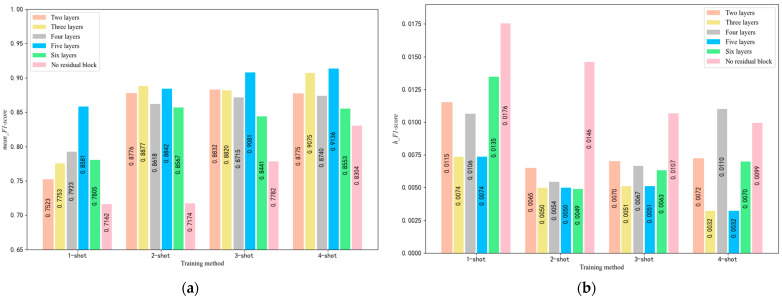
(**a**) *mean_F*1*-score* for different numbers of residual block layers under four training methods. (**b**) *h_F*1*-score* for different numbers of residual block layers under four training methods.

Prof. Kaiming He’s study suggested that the number of layers in the residual block should be at least two, as a single layer would not be meaningful [47]. To examine the suitability of using five layers of residual blocks, we conducted an experiment where we varied the number of layers in the embedding module and selected records between 700 and 1000 epochs to obtain the *mean_F*1*-score* and *h_F*1*-score*. Additionally, we replaced each residual block with five concatenated convolutional layers to investigate the necessity of using residual blocks. The resulting histogram is shown in Figure 9.

To provide a deeper analysis of the results in Figure 9, we observe that the 5-layer residual block consistently outperforms other configurations across all few-shot tasks (4-way 1-shot to 4-way 4-shot). Specifically, for the 4-way 4-shot task, the mean_F1-score peaks at 0.931 with an h_F1-score of 0.008, indicating not only high accuracy but also exceptional stability. This optimal performance is attributed to the residual blocks’ ability to mitigate gradient vanishing while enabling sufficient depth for feature abstraction. In contrast, fewer layers result in lower mean_F1-scores due to inadequate hierarchical representation learning. Conversely, while not tested beyond five layers, excessive depth (implied by the trend) could increase computational latency and overfitting risks, as seen in the marginal decline in stability for non-residual configurations. Additionally, the reduced h_F1-score for 5-layer blocks underscores their robustness to input variations, which is critical for small-sample COPD detection where data noise is prevalent. This analysis confirms that a 5-layer residual design achieves an optimal trade-off between model complexity and generalization, directly supporting our architectural choice for SE-RelationNet.

### 5.3. Control Experiments with Other Models

We chose SiameseNet and ProtoNet as the control networks for SE-RelationNet, which are described below.

**Figure 10:**
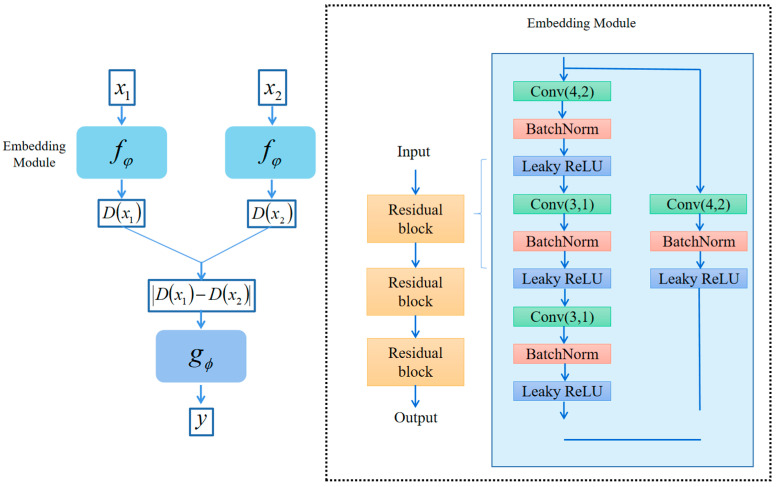
The structure of the SiameseNet.

The SiameseNet is a basic one-shot learning method that has been adapted to also handle few-shot problems with *K* > 1. Its structure is shown in Figure 10. The embedding module outputs a feature vector, which is used to compute the similarity between a pair of samples in the query set and support set. This is achieved by averaging the feature vectors of the support set samples and subtracting from the feature vectors of the query set samples. The absolute values are then input to the metrics module, which consists of two fully connected layers, and outputs a number between 0 and 1 representing the similarity of the two samples.

**Figure 11:**
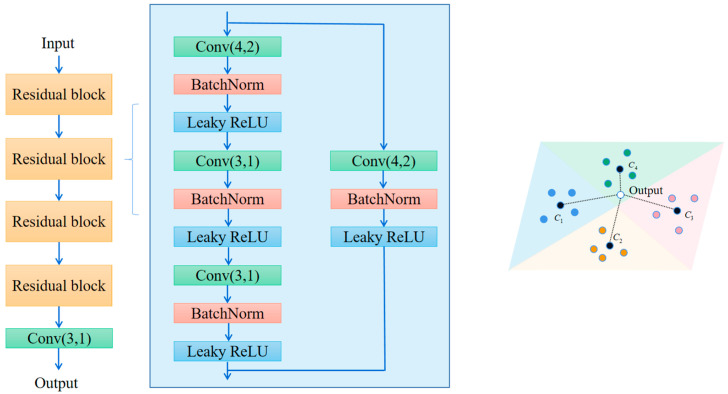
The structure of the ProtoNet.

ProtoNet is a few-shot learning method, with the structure shown in Figure 11. *K* samples of each class in the support set are projected into the Euclidean space by the embedding module, and the average value is taken as the prototypical vector of that class. Samples in the query set are projected by the embedding module, and the Euclidean distance from the prototypical vector of each class is calculated. Finally, the Softmax function is used to evaluate the probability that the samples belong to each category.

**Figure 12:**
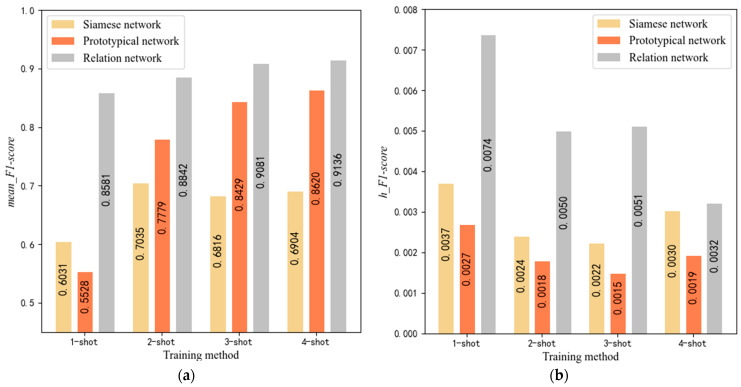
(**a**) *mean_F*1*-score* for SE-RelationNet and its control networks after training in four ways. (**b**) *h_F*1*-score* for SE-RelationNet and its control networks after training in four ways.

The learning rates for SiameseNet, ProtoNet, and SE-RelationNet are 0.0001, 0.00001, and 0.0001, respectively. We selected the *F*1*-score* recorded between 700 and 1000 training rounds and calculated the *mean_F*1*-score* and *h_F*1*-score*. The results are shown in Figure 12.

Figure 12 shows that in the one-shot learning task, the *mean_F*1*-score* of both the SiameseNet and the ProtoNet is less than 0.7, while the SE-RelationNet is higher than 0.8. The lower *h_F*1*-score* of the SiameseNet and the ProtoNet indicates that they have higher stability; in the one-shot learning task, the SE-RelationNet has higher accuracy and lower stability. In the few-shot learning task with *K* > 1, the SE-RelationNet has higher accuracy and lower stability, but its stability gets significantly improved as *N* increases.

**Figure 13:**
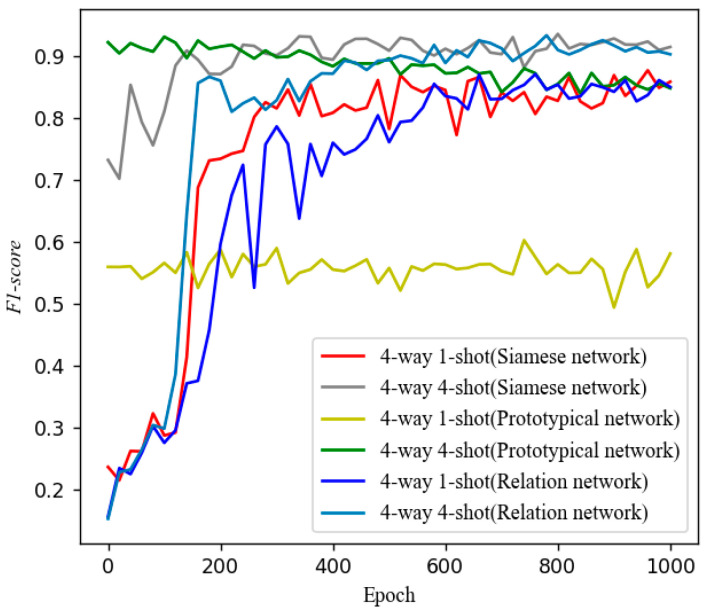
Trend of *F1-score* with epoch using different training methods for three models.

The trend of *F*1*-score* with epoch for the three models is plotted in Figure 13 under 4-way 1-shot and 4-way 4-shot training methods. Regardless of the training method, the ProtoNet can reach convergence after very few training rounds, while the SiameseNet and the SE-RelationNet need more than 100 training rounds to converge.

## 6. Conclusions

In this paper, we propose an electronic nose based on SE-RelationNet for identifying COPD patients by analyzing their breath samples when the number of labeled samples is limited. SE-RelationNet consists of an embedding module and a metric module, and we conducted several ablation experiments on its structure to optimize its performance. The results showed that using a 5-layer residual block and a BiGRU block with one hidden layer as the metric module achieved the highest accuracy and stability. Compared to SiameseNet and ProtoNet, our model demonstrated superior performance, achieving a mean accuracy of 93.3% in 4-way 4-shot tasks and outperforming SiameseNet by 15.2% in F1-score under one-shot conditions.

However, two limitations require attention:

(1) Cross-device generalizability: While SE-RelationNet reduces sensor dependency (Section 2.1 and Section 2.2), performance fluctuations occur when meta-training/meta-testing sensor arrays differ significantly (*h_accuracy* ≤ 0.010 in Table 7).
(2) Clinical-scale validation: Current validation used curated public datasets. Real-world clinical trials with diverse patient cohorts are needed to assess robustness against comorbidities like asthma or pneumonia.

Future work will focus on:

(1) Extending the model to multi-class COPD severity detection (mild/moderate/severe) using VOC profiles, leveraging the COPD-LUCSS risk correlation.
(2) Integrating lung cancer biomarkers (e.g., aldehyde/ketone signatures) into sensor arrays for joint screening.
(3) Addressing the above limitations through hybrid sensor-fusion algorithms and multi-center clinical trials.

SE-RelationNet’s relocatability enables researchers to build the e-nose using common sensor arrays, requiring only minimal breath samples (as few as one per class) for COPD detection. This facilitates early lung cancer risk stratification in high-risk COPD populations, ultimately promoting accessible point-of-care diagnostics. To translate COPD discrimination into lung cancer risk stratification, we propose:

(1) Multi-class COPD subtype detection—Extend SE-RelationNet to classify COPD severity (mild/moderate/severe) using VOC profiles, leveraging the established COPD-LUCSS risk correlation.
(2) Biomarker integration—Incorporate lung cancer-specific biomarkers (e.g., aldehyde/ketone signatures) into the sensor array, enabling simultaneous COPD/lung cancer screening.
(3) Hybrid risk modeling—Develop algorithms combining COPD subtypes, biomarkers, and clinical factors to generate quantifiable risk scores.
(4) Prospective validation—Conduct multi-center trials to validate stratification efficacy prior to clinical deployment.
(5) This framework bridges the gap between our technology and actionable cancer-prevention strategies.

## Figures and Tables

**Table 1:** Comparison of COPD detection methods. Accuracy, speed, cost, complexity, personnel requirement, and invasiveness are key factors differentiating traditional methods from electronic nose technology.

Detection Method	Accuracy	Speed	Cost	Complexity	Personnel Requirement	Invasive?	Key Limitations
Gas Chromatography–Mass Spectrometry (GC-MS) [9,10]	High	Slow (hrs)	High	High	Specialized	No	Time-consuming, expensive equipment and maintenance, complex sample prep and analysis
Spirometry [11,12,13]	Moderate	Moderate	Low– Mod	Moderate	Trained	No	Effort-dependent, may miss early disease, requires patient cooperation
Sputum Cytometry	Variable	Moderate	Mod	Moderate	Trained	No	Sample variability, requires specialized staining/analysis
Chest Radiography (X-ray) [14]	Low– Mod	Fast	Low– Mod	Low	Trained (interpretation)	No	Low sensitivity for early COPD, limited specificity (other lung conditions look similar)
Fluoroscopic Bronchoscopy	High	Slow	High	High	Specialist	Yes	Invasive, requires sedation/anesthesia, risk of complications, expensive
Electronic Nose (E-nose) [15]	High (Emerging)	Fast (mins)	Lower (Potential)	Lower	Minimal Training	No	Requires algorithm development/validation, sensor drift/calibration needs

**Table 2:** Location, type, and sensitive gas of selected sensors in meta-training set ([*x*, *y*] denotes the *y*th sensor of the *x*th group).

No.	Location	Type	Mean Contribution	Slope Contribution	Contribution Score	Sensitive Gas
1	<4,4>	TGS2600	0.5131	0.6426	0.5778	Hydrogen, carbon, monoxide
2	<5,2>	TGS2612	0.9244	0.7229	0.8236	Methane, propane, butane
3	<5,3>	TGS2610	0.5159	0.4822	0.4991	Propane
4	<5,4>	TGS2600	0.8593	1.0441	0.9517	Hydrogen, carbon, monoxide
5	<5,5>	TGS2602	0.4782	0.5130	0.5130	Ammonia, H_2_S, volatile organic compounds (VOC)
6	<5,6>	TGS2602	0.5004	0.5228	0.5116	Ammonia, H_2_S, VOC
7	<5,7>	TGS2620	0.4925	0.5665	0.5295	Carbon, monoxide, combustible gases, VOC
8	<5,8>	TGS2620	0.5246	0.5920	0.5583	Carbon, monoxide, combustible gases, VOC

**Table 3:** Correspondence of gas class, molecular formula, concentration, and sample size in meta-training set.

Class	Molecular Formula	Concentration (ppm)	Number of Gas Samples
Acetaldehyde	$C_{2}H_{4}O$	500	1800
Acetone	$C_{3}H_{6}O$	2500	1800
Ammonia	${\mathrm{NH}}_{3}$	10,000	1800
Benzene	$C_{6}H_{6}$	200	1800
Butanol	$C_{4}H_{9}\mathrm{OH}$	100	1500
Carbon monoxide	C O	4000	1571
Carbon monoxide	C O	1000	449
Ethylene	$C_{2}H_{4}$	500	1800
Methane	${\mathrm{CH}}_{4}$	1000	1800
Methanol	${\mathrm{CH}}_{4}O$	200	1800
Toluene	$C_{7}H_{8}$	200	1800

**Table 4:** Correspondence of gas classes and their respective quantities in meta-testing set.

Class	The Number of Samples
COPD	40
Smokers	8
Control	20
Air	10

**Table 5:** Parameter setting of SE-RelationNet.

Parameter Names	Parameter Values
Optimizer	Adam
Loss function	Mseloss
Training epochs	1001
Testing epochs	50
Batch num per class during training	20
BiGRU’s hidden layers	1
Learning rate	0.0001
Seed	512
Dropout	0.3
ratio	16

**Table 6:** Confusion matrix.

		Reference	
		Positive	Negative
Prediction	Positive	TP	FP
	Negative	FN	TN

**Table 7:** Model performance metrics under four training methods.

	*mean_accuracy*	*h_accuracy*	*mean_F*1*-score*	*h_F*1*-score*
4-way 1-shot	0.858	0.010	0.852	0.011
4-way 2-shot	0.896	0.005	0.890	0.006
4-way 3-shot	0.922	0.008	0.919	0.008
4-way 4-shot	0.933	0.007	0.931	0.008

**Table 8:** Mean_F1-score obtained by four training methods when using six different modules.

	Group 1	Group 2	Group 3	Group 4	Group 5	Group 6	Group 7
4-way 1-shot	0.852	0.845	0.842	0.816	0.823	0.808	0.819
4-way 2-shot	0.890	0.869	0.874	0.893	0.902	0.855	0.880
4-way 3-shot	0.919	0.904	0.919	0.919	0.915	0.878	0.893
4-way 4-shot	0.931	0.915	0.915	0.925	0.926	0.882	0.905

## Data Availability

The data used is from public datasets, and the code will be made public after the paper is accepted.
